# Psychological Distress, Fear and Coping Strategies During the Second and Third Waves of the COVID-19 Pandemic in Southern Germany

**DOI:** 10.3389/fpsyt.2022.860683

**Published:** 2022-04-25

**Authors:** Mohamed Elsayed, Carlos Schönfeldt-Lecuona, Xenia Anna Welte, Khaled Tarek Dardeer, Manar Ahmed Kamal, Ramy Abdelnaby, Markus A. Rudek, Evelyne Riedel, Michael Denkinger, Maximilian Gahr, Bernhard J. Connemann, Sheikh M. Alif, Biswajit Banik, Wendy Cross, Muhammad Aziz Rahman

**Affiliations:** ^1^Department of Psychiatry and Psychotherapy III, University of Ulm, Ulm, Germany; ^2^Geriatric Center Ulm (GZU), Ulm, Germany; ^3^Faculty of Medicine, Cairo University, Cairo, Egypt; ^4^Faculty of Medicine, Benha University, Benha, Egypt; ^5^Department of Neurology, RWTH Aachen University, Aachen, Germany; ^6^Division of Nephrology, University of Heidelberg, Heidelberg, Germany; ^7^Center of Psychiatry (ZfP), Biberach, Germany; ^8^Agaplesion Bethesda Clinic, Geriatric Research Unit Ulm University, Ulm, Germany; ^9^School of Public Health and Preventive Medicine, Monash University, Melbourne, VIC, Australia; ^10^School of Health, Federation University Australia, Berwick, VIC, Australia; ^11^Faculty of Public Health, Universitas Airlangga, Surabaya, Indonesia

**Keywords:** COVID-19, psychological distress, fear, cross-sectional survey, coping, mental health, Germany

## Abstract

**Background:**

The COVID-19 pandemic has imposed enormous psychological discomfort and fear across the globe, including Germany.

**Objectives:**

To assess the levels of COVID-19 associated psychological distress and fear amongst Southern German population, and to identify their coping strategies.

**Methods:**

A cross-sectional survey using an online questionnaire was conducted in healthcare and community settings in the region of Ulm, Southern Germany. Assessment inventories were the Kessler Psychological Distress Scale (K-10), the Brief Resilient Coping Scale (BRCS), and the Fear of COVID-19 Scale (FCV-19S), which were valid and reliable tools.

**Results:**

A total of 474 Individuals participated in the study. The mean age was 33.6 years, and 327 (69%) were females. Most participants (*n* = 381, 80.4%) had high levels of psychological distress, whereas only 5.1% had high levels of fear, and two-thirds of participants showed higher levels of coping. Moderate to very high levels of psychological distress were associated with being female, living alone, distress due to employment changes, experiencing financial impact, having multiple co-morbidities, being a smoker, increased alcohol use over the previous 6 months, contact with COVID-19 cases and healthcare providers for COVID-19-related stress. Individuals who were ≥60 years, lived with non-family members, had co-morbidities and visited a healthcare provider had higher levels of fear. Higher levels of education and income showed better coping amongst participants.

**Conclusion:**

Psychological distress was very high during the COVID-19 pandemic in Germany and associated with low levels of coping. This study identified vulnerable groups of people, who should be given priorities for addressing their health and wellbeing in future crisis periods.

## Introduction

The coronavirus disease (COVID-19) has spread into 222 countries and territories worldwide and the World Health Organization (WHO) declared a global public health emergency on 30 January 2020 (1). As of 23 November 2021, Germany reported more than five million confirmed cases and almost 100,000 deaths from COVID-19 (2). This led to enact public health measures by the Government such as physical distancing, canceling large gatherings, imposing travel restrictions and lockdown in large cities, ensuring obligatory quarantine for positive cases, primary close contacts, along with closing of educational institutions. The lockdown also resulted in the closure of many small businesses, and the unemployment rate increased to 4.1% in summer 2020 compared to 3.1% just before the pandemic (3). Ongoing restrictions also impacted on the physical and mental health of the population, especially older adults with multiple comorbidities (4). Ongoing social isolation and uncertainty of further COVID-19 pandemic waves could potentially trigger long-term mental disorders (5).

Furthermore, Unemployment and social isolation were associated with risky behaviors such as increased tobacco and alcohol consumption (6). Lockdown measures and social distancing restrictions caused a shift to telehealth facilities (7). Previous studies showed that healthcare workers engaged in the diagnosis and management of COVID-19 patients were more prone to psychological distress and various mental disorders, such as depression, anxiety, anger, fear of spreading the infection to their relatives, friends, or colleagues (8, 9).

Studies from several countries around the world including Germany found that the pandemic caused higher psychological distress, anxiety, and depression amongst a large proportion of community members (10–16). Studies also showed that depression, stress, and anxiety during the pandemic triggered sleep disorders and increased consumption of tobacco and alcohol (17). However, with increased vaccination rates and easing of restrictions, impacts may change during the current pandemic waves. Although there are previously published studies that assessed anxiety, fear and distress amongst community members and healthcare workers in Germany during the COVID-19 pandemic, the existing evidence lacks a full understanding of the impacts of the pandemic on mental health and coping strategies amongst the public in Germany and identification of the relevant predictors. Therefore, this study aimed to assess the levels of psychological distress, fear of the COVID-19 disease, and coping strategies among a wide range of population in Germany; it also aimed to identify critical factors associated with those outcomes. The population subgroups who were at higher risk of developing poor mental health outcomes would be identified in this study, which would enable the policymakers to optimize psychosocial interventions targeted to those vulnerable groups of population and guide resource planning to avoid long-term mental health impacts.

## Materials and Methods

### Study Design and Setting

A cross-sectional study was conducted according to The Strengthening the Reporting of Observational Studies in Epidemiology (STROBE) Statement Checklist (18). This study was a part of a large study involving 17 countries and was led by the last author (10). Participants were informed about the study using social media and received the link of the questionnaire through social media or by emails. In addition, Quick Response (QR) codes were used on professional posters in outpatient clinics to inform patients about the study and invite participation. Data were collected from healthcare settings, including General Practices, hospitals, allied health professionals, and community settings, using a structured self-administered online questionnaire (10, 13, 14, 19).

### Study Population

Adult participants who were ≥18 years old with the capacity to respond to an online questionnaire in German language were included in three main groups: (a) patients who attended a healthcare setting, either for face-to-face or telehealth consultation in the last 4 weeks irrespective of respiratory/COVID-19 symptoms, (b) healthcare workers (full time, part-time or casual) who have been in contact with patients in the last 4 weeks in a healthcare setting (doctor, nurses, allied health professionals, technicians, patient service attendants, receptionists, etc.) irrespective of caring for respiratory/COVID-19 patients, and (c) community members who did not consult any healthcare provider in the last 4 weeks.

### Data Collection

An online link to the web-based questionnaire was developed using Google forms to collect data from February to April 2021 during the second and third waves of the COVID-19 pandemic. According to Robert Koch Institute, the second German wave began in October 2020 (https://www.shorturl.at/shortener.php), while the third wave started in March 2021 (https://www.shorturl.at/loqHP). Initially, there was a screening question related to age to confirm eligibility; subsequently data were collected on: (a) socio-demographics as age, gender, location of residence, marital status, living conditions (alone or with families), the highest level of education, country of birth; (b) profession as a primary occupation, the impact of COVID-19 on occupation, identification as a frontline healthcare worker; (c) self-reported comorbidities as hypertension, cardiovascular diseases, chronic respiratory diseases, diabetes, cancer; (d) behavioral risk factors as current smoking, alcohol intake; (e) health service utilization (in the last 4 weeks) as consultation with a healthcare provider for any symptom, admission to the hospital including reasons for admission; (f) exposure and contact history of COVID-19, test and diagnosis of COVID-19, close contact, isolation and quarantine status; (g) psychological impact measured by the Kessler Depression Scale (K-10) (20), and fear measured by the Fear of COVID-19 Scale (FCV) (21); (h) coping strategies measured by the Brief Resilient Coping Scale (BRCS) (22); and (j) access to mental health resources (in the last four weeks) (see Appendix 1).

### Study Tools

#### Kessler Psychological Distress Scale (K10)

The K10 scale is a 10-item self-rated questionnaire that measures distress based on depressive and anxiety symptoms. Each item has five possible answers (none of the time = 1, a little of the time = 2, some of the time = 3, most of the time = 4, all of the time = 5) allowing for a total score of 50. A score of 10–15 is likely to be well, 16–29 is medium risk for anxiety or depression and 30–50 is high risk for anxiety or depressive symptoms (20). Cronbach's alpha for this tool was 0.902, which was satisfactory.

#### Fear of COVID-19 Scale (FCV-19S)

The FCV-19S is a seven-item scale that assesses fear of COVID-19 among the general population. Each item has five possible answers (strongly disagree = 1 disagree = 2 neutral = 3 agree = 4 strongly agree = 5) allowing for a maximum score of 35, a score of 7-22 is considered low fear and 23–35 is considered high fear of COVID-19 (21). Cronbach's alpha for this tool was 0.82, which was satisfactory.

#### Brief Resilient Coping Scale (BRCS)

The BRCS is a 4-item scale that measures a psychological well-being construct: resilience. Each item is a 5-point response (does not describe me at all = 1, does not describe me = 2, neutral = 3, describes me = 4, describes me very well = 5). The maximum possible score is 20, and it is categorized into low resilience (score 4–13), medium resilience (score 14–16) and high resilience (Score 17–20) (22). Cronbach's alpha for this tool was 0.758, which was satisfactory.

### Sample Size Calculation

All participants fulfilling the inclusion criteria were invited to participate. Considering Germany's population of 84 million according to World Population Prospects (23), the prevalence of lifetime mental health issues amongst Germans was 31.1% (24), at 95% confidence intervals, margin of error (5%), and 80% power, the required sample size was 329. The sample size was calculated using Open Epi Info software version 7.2. Convenient sampling was used to recruit the study participants by following snowball sampling.

### Ethics

Ethical approval was obtained from the Research Ethical Committee (REC) of the Ulm University (Ethical Approval Number 448/20 – FSt/Sta).

### Statistical Analysis

International Business Machines Corporation (IBM) Statistical Package for the Social Sciences (SPSS) statistics software version 25 was used for data analysis. Descriptive analyses were conducted and followed by inferential analyses. Continuous variables were presented as mean ± standard deviation (SD), while categorical variables were presented as numbers and percentages. Internal consistency of the instruments was calculated using Cronbach's alpha. The study outcomes were categorized into binary variables as follows: K-10 score was categorized into low (score 10–15) and moderate to very high (score 16–50), FCV-19S score was categorized into low (score 7–21) and high (score 22–35) and BRCS score was defined into low (score 4–13) and medium to high (score 14–20) resilient copers. Univariate and multivariate logistic regression (adjusted for age, gender, born in Germany, living status, employment status, level of education) were performed to explore the association between population characteristics and the study's outcomes. Odds ratios (ORs), adjusted odds ratios (AORs) and 95% confidence intervals (95% CIs) were obtained. Firth logistic regression with penalized maximum likelihood was used for fear of COVID-19 outcome as the number of events was relatively low for the number of adjusted variables. To measure the association between distress, fear and coping, partial rank correlation was conducted on the overall score as a continuous variable for each scale. This was done after controlling for potential confounding factors (age, gender, born in Germany, living status, employment status and level of education). P values less than 0.05 were considered statistically significant.

## Results

### Population Characteristics

A total of 474 people participated in the study. The mean age of the participants was 33.6 (13.3) years, and 327 (69%) were females. Most of them were born in Germany (89.9%, n=426), and around half lived with family members (48.5%, *n* = 230). Two-thirds (62.4%, *n* = 296) of the participants had a source of income during the pandemic, and only 2.1% (*n* = 10) had their jobs affected by the pandemic. Half of the participants (57%, *n* = 270) reported change in the employment situation, and half of them (49.4%, *n* = 196) had higher perceived distress due to that change. About half participants (47.3%, *n* = 224) self-identified as essential service workers and 54.6% (*n* = 259) reported being healthcare workers. Only 7.4% (*n* = 35) participants reported having psychiatric or mental health issues, although a quarter of the participants (24.1%, *n* = 114) perceived their mental health status as poor to fair. Table 1 shows the characteristics of included participants, and Tables 2–**4** shows multivariate analyses of psychological distress, fear, and coping.

**Table 1:** Baseline demographic characteristics of the participants (*N* = 474).

**Characteristic**	**No**.	**%**
**Age**	**474**	
Mean (± SD)	33.6 ± 13.32	
Age groups	474	
18–29	232	48.9
30–59	220	46.4
>60	22	4.6
Gender	474	
Female	327	69.0
Born in Germany	474	
Yes	426	89.9
Living status	474	
Live with family members	230	48.5
Live with non-family members	145	30.6
Live alone	99	20.9
Highest educational/vocational qualification	474	
Secondary/Higher Secondary/Grade 7 to 12	188	39.7
Certificate/Diploma/Trade qualifications	83	17.5
Bachelor/Masters/PhD	203	42.8
Current employment condition	474	
Unemployed/Housewife/Homemaker/Home duties (No source of income)	168	35.4
Jobs affected by COVID-19 (lost job/working hours reduced/afraid of job loss)	10	2.1
Have an income source (employed/Government benefits)	296	62.4
Perceived distress due to change of employment status	397	
A little to none	201	50.6
Moderate to a great deal	196	49.4
Improved working situation due to change of employment status	406	
A little to none	352	86.7
Moderate to a great deal	54	13.3
Self-identification as frontline or essential service worker	474	
Yes	224	47.3
Self-identification as a healthcare worker	474	
Yes, doctor	64	13.5
Yes, nurse	18	3.8
Yes, another healthcare worker	177	37.3
COVID-19 impacted the financial situation	474	
Yes, impacted positively	43	9.1
Yes, impacted negatively	67	14.1
Affected by the change in a financial situation	474	
Not at all	198	41.8
Unsure	53	11.2
Somewhat	130	27.4
A great extent	39	8.2
Co-morbidities	474	
Psychiatric/Mental health problem	35	7.4
Other co-morbidities^*^	102	21.5
Co-morbidities	474	
Single co-morbidity	98	20.7
Multiple co-morbidities	43	9.1
Perceived status of own mental health	474	
Good to Excellent	360	75.9
Poor to Fair	114	24.1
Smoking	474	
Ever smoker (Daily/Nondaily/Ex)	59	12.4
Increased smoking over the last 6 months	59	
Yes	24	41.7
Current alcohol drinking (last 4 weeks)	474	
Yes	200	42.2
Increased alcohol drinking over the last 6 months	200	
Yes	46	23
Contact with known/suspected case of COVID-19	474	
Unsure	51	10.8
Yes, I had indirect contact	78	16.5
Yes, provided direct care	105	22.2
Experience related to COVID-19 pandemic	474	
No known exposure to COVID-19	334	70.5
Treated in hospital / Ordered to quarantine/ Tested positive / Lived with someone who had COVID-19	123	25.9
Traveled overseas and had to quarantine	17	3.6
Self-identification as a patient (visited a healthcare provider in the last 6 months)	474	
Yes	224	47.3
If yes, which type of healthcare did you use? (Multiple responses)	268	
Visit a primary care physician or health care professional	184	68.7
Telehealth consultation (online or by phone) with a general practitioner, specialist, or health professional	10	3.7
I was tested for COVID-19 at a special test site	42	15.7
Hospital emergency room	8	3.0
I was in a hospital for other reasons	24	9.0
Healthcare service used to overcome COVID-19 related stress in the last 6 months	474	
Yes	25	5.3
If yes, which type of healthcare did you receive? (Multiple responses)	61	
Consulted a primary care physician	26	42.6
Consulted a psychologist	17	27.9
Consulted a psychiatrist	4	6.6
Used specialty mental health services (hospital, community mental health services, inpatient mental health services)	2	3.3
Used mental health resources (pamphlets, brochures, leaflets, and books provided by mental health staff and distributed at hospital)	3	4.9
Mental health resources used and available through media (methods and techniques of psychological support provided by psychologists through online media, television news, or various online and social networking platforms)	5	8.2
Mental health support services used (including mental health program)	4	6.6

*Data are presented as the mean and standard deviation (SD) or number (n) and percentage (%)*.

*COVID-19, Coronavirus Disease 19; K-10, Kessler Depression Scale; FCV, Fear of Coronavirus-19 Scale; BRCS, Brief Resilient Coping Scale*.

* *Cardiac diseases/ Stroke/ Hypertension/ Hyperlipidaemia/ Diabetes/ Cancer/ Chronic respiratory illness*.

**Table 2:** Factors associated with high psychological distress among the study population (based on K10 scoring).

**Characteristics**	**Low distress**		**High distress**		**Unadjusted analysis**			Adjusted analysis^*^				
	**n**	**%**	**n**	**%**	**p**	**ORs**	**95% CIs**		**p**	**AORs**	**95% CIs**	
Age groups	93		381									
18–29	23	9.9	209	90.1	Ref				Ref			
30–59	58	26.4	162	73.6	**<0.001**	**0.31**	**0.18**	**0.52**	**0.029**	**0.41**	**0.18**	**0.91**
>60	12	54.5	10	45.5	**<0.001**	**0.09**	**0.04**	**0.24**	**0.000**	**0.10**	**0.03**	**0.33**
Gender	93		381									
Male	51	34.7	96	65.3	Ref				Ref			
Female	42	12.8	285	87.2	**<0.001**	**3.61**	**2.26**	**5.76**	**0.000**	**4.04**	**2.41**	**6.77**
Born in the same country of residence	93		381									
No	7	14.6	41	85.4	Ref				Ref			
Yes	86	20.2	340	79.8	0.357	1.48	0.64	3.42	0.055	0.41	0.16	1.02
Living status	93		381									
Live with family members	63	27.4	167	72.6	Ref				Ref			
Live with non-family members	16	11.0	129	89.0	**<0.001**	**3.04**	**1.68**	**5.51**	0.530	1.32	0.55	3.17
Live alone	14	14.1	85	85.9	**0.011**	**2.29**	**1.21**	**4.32**	**0.041**	**2.05**	**1.03**	**4.01**
Highest educational/vocational qualification	93		381									
Secondary/Higher Secondary/Grade 7 to 12	21	11.2	167	88.8	Ref				Ref			
Certificate/Diploma/Trade qualifications	23	27.7	60	72.3	**0.001**	**0.33**	**0.17**	**0.64**	0.094	0.49	0.21	1.13
Bachelor/Masters/PhD	49	24.1	154	75.9	**0.001**	**0.40**	**0.23**	**0.69**	0.317	0.68	0.32	1.44
Current employment condition	93		381									
Unemployed/Housewife/Home maker/Home duties (No source of income)	22	13.1	146	86.9	Ref				Ref			
Jobs affected by COVID-19 (lost job/working hours reduced/afraid of job loss)	1	10.0	9	90.0	0.778	1.36	0.16	11.23	0.272	3.56	0.37	34.23
Have an income source (employed/Government benefits)	70	23.6	226	76.4	**0.007**	**0.49**	**0.29**	**0.82**	0.522	1.27	0.61	2.63
Perceived distress due to change of employment status	68		329									
A little to none	47	23.4	154	76.6	Ref				Ref			
Moderate to a great deal	21	10.7	175	89.3	**0.001**	**2.54**	**1.45**	**4.44**	**0.001**	**2.85**	**1.54**	**5.27**
Improved working situation due to change of employment status	72		334									
A little to none	65	18.5	287	81.5	Ref				Ref			
Moderate to a great deal	7	13.0	47	87.0	0.327	1.52	0.66	3.52	0.735	1.17	0.48	2.85
Self-identification as a frontline or essential service worker	93		381									
No	44	17.6	206	82.4	Ref				Ref			
Yes	49	21.9	175	78.1	0.243	0.76	0.48	1.20	0.656	1.15	0.63	2.08
Self-identification as a healthcare worker	93		381									
No	37	17.2	178	82.8	Ref				Ref			
Yes, doctor	16	25.0	48	75.0	0.166	0.62	0.32	1.22	0.199	1.80	0.73	4.42
Yes, nurse	2	11.1	16	88.9	0.51	1.66	0.37	7.54	0.385	2.08	0.40	10.79
Yes, other healthcare worker	38	21.5	139	78.5	0.287	0.76	0.46	1.26	0.538	0.82	0.43	1.55
COVID-19 impacted financial situation	93		381									
No impact	80	22.0	284	78.0	Ref				Ref			
Yes, impacted positively	6	14.0	37	86.0	0.228	1.74	0.71	4.26	0.286	1.74	0.63	4.78
Yes, impacted negatively	7	10.4	60	89.6	**0.035**	**2.41**	**1.06**	**5.49**	**0.006**	**3.61**	**1.45**	**9.00**
Affected by the change in financial situation	79		341									
Not at all	55	27.8	143	72.2	Ref				Ref			
Unsure	6	11.3	47	88.7	**0.017**	**3.01**	**1.22**	**7.45**	**0.046**	**2.78**	**1.02**	**7.61**
Somewhat	16	12.3	114	87.7	**0.001**	**2.74**	**1.49**	**5.04**	**0.001**	**3.34**	**1.67**	**6.68**
A great extent	2	5.1	37	94.9	**0.008**	**7.12**	**1.66**	**30.35**	**0.009**	**7.51**	**1.66**	**33.94**
Co-morbidities	93		381									
No	66	19.6	271	80.4	Ref				Ref			
Psychiatric/Mental health problem	3	8.6	32	91.4	0.123	2.60	0.77	8.74	0.095	2.97	0.83	10.64
Other co-morbidities^*^	24	23.5	78	76.5	0.388	0.79	0.47	1.35	0.622	0.86	0.48	1.56
Co-morbidities	93		381									
No	64	19.2	269	80.8	Ref				Ref			
Single co-morbidity	24	24.5	74	75.5	0.256	0.73	0.43	1.25	0.400	0.77	0.42	1.42
Multiple co-morbidities	5	11.6	38	88.4	0.232	1.81	0.68	4.78	**0.042**	**3.12**	**1.04**	**9.33**
Perceived status of own mental health	93		381									
Good to Excellent	93	25.8	267	74.2	Ref							
Poor to Fair	0	0.0	114	100.0	**0.002**	**80.04**	**4.93**	**1,300.40**	**No. is too low to estimate**			
Smoking	93		381									
Never smoker	88	21.2	327	78.8	Ref				Ref			
Ever smoker (Daily/Nondaily/ Ex)	5	8.5	54	91.5	**0.027**	**2.91**	**1.13**	**7.49**	**0.007**	**4.13**	**1.48**	**11.58**
Increased smoking over the last 6 months	5		54									
No	5	14.3	30	85.7	Ref							
Yes	0	0.0	24	100.0	0.14	8.83	0.46	167.70	0.112	6.69	0.70	886.78
Current alcohol drinking (last 4 weeks)	93		381									
No	51	18.6	223	81.4	Ref				Ref			
Yes	42	21.0	158	79.0	0.518	0.86	0.55	1.36	0.958	1.10	0.61	1.70
Increased alcohol drinking over the last 6 months	42		158									
No	39	25.3	115	74.7	Ref				Ref			
Yes	3	6.5	43	93.5	**0.011**	**4.86**	**1.43**	**16.55**	**0.019**	**4.77**	**1.29**	**17.61**
Contact with known/suspected case of COVID-19	93		381									
No	58	24.2	182	75.8	Ref							
Unsure	10	19.6	41	80.4	0.486	1.31	0.62	2.77	0.542	1.30	0.56	3.02
Yes, had indirect contact	10	12.8	68	87.2	**0.037**	**2.17**	**1.05**	**4.48**	**0.043**	**2.26**	**1.03**	**4.98**
Yes, provided direct care	15	14.3	90	85.7	**0.041**	**1.91**	**1.03**	**3.56**	**0.017**	**2.33**	**1.17**	**4.68**
Experience related to COVID-19 pandemic	93		381									
No known exposure to COVID-19	80	24.0	254	76.0	Ref				Ref			
Treated in hospital / Ordered to quarantine/ Tested positive / Lived with someone who had Covid-19	11	8.9	112	91.1	**0.001**	**3.21**	**1.64**	**6.26**	**0.009**	**2.59**	**1.26**	**5.30**
Traveled overseas and had to quarantine	2	11.8	15	88.2	0.260	2.36	0.53	10.55	0.428	1.89	0.39	9.06
Self-identification as a patient (visited a healthcare provider in the last 6 months)	93		381									
No	54	21.6	196	78.4	Ref				Ref			
Yes	39	17.4	185	82.6	0.252	1.31	0.83	2.07	0.271	1.34	0.80	2.26
Level of fear of COVID-19 (FCV- 19S categories)	93		381									
Low (score 7–21)	91	20.2	359	79.8	Ref				Ref			
High (score 22–35)	2	8.3	22	91.7	0.170	2.79	0.64	12.08	0.142	3.26	0.67	15.74
Level of coping (BRCS categories)	93		381									
Low resilient coping (score 4– 13)	24	15.2	134	84.8	Ref				Ref			
Medium to high resilient coping (score 14–20)	69	21.8	247	78.2	0.087	0.64	0.39	1.07	0.097	0.61	0.34	1.09
Healthcare services used to overcome COVID-19 related stress in the last 6 months	93		381									
No	93	20.7	356	79.3	Ref				Ref			
Yes	0	0.0	25	100.0	**0.004**	**13.37**	**1.84**	**1,702.70**	**0.006**	**13.58**	**1.77**	**1,752.73**

*Data are presented as number (n) and percentage (%). P ≤ 0.05 were considered statistically significant*.

*ORs, Odds Ratio; AOR, Adjusted Odds Ratio; CI, Confidence Interval; Ref, Reference category; COVID-19, Coronavirus Disease 19; K-10, Kessler Depression Scale; FCV, Fear of Coronavirus-19 Scale; BRCS, Brief Resilient Coping Scale*.

* *Adjusted for Age, Gender, born in Germany, living status, employment status and level of education*.

*Bold indicated statistical significance*.

### Psychological Distress

After adjusting for potential confounders, multivariate analyses showed that being a female, living alone, those with distress due to employment change, worsened financial situation, having multiple co-morbidities, smoking, increased alcohol consumption over the last 6 months, contact with COVID-19 case whether direct or indirect, direct experience of COVID-19 and healthcare use to overcome pandemic stress in the last 6 months were associated with moderate to very high levels of psychological distress (Table 2).

### Fear of COVID-19

Multivariate logistic regression showed that being over 60, living with non-family members, those having a diploma or a trade qualification, those with single or multiple comorbidities, perceived mental health status as poor to fair, direct experience of COVID-19, visiting a health care provider in the past 6 months and using healthcare service to overcome pandemic related stress in the last 6 months were associated with higher levels of fear of COVID-19 (Table 3).

**Table 3:** Factors associated with high levels of fear of COVID-19 among the study population (based on FCV-19S scoring).

**Characteristics**	**Low levels of fear**		**High levels of fear**		**Unadjusted analyses**			**Adjusted analysis**				
	**n**	**%**	**n**	**%**	**p**	**ORs**	**95% CIs**		**p**	**AORs**	**95% CIs**
Age groups	450		24									
18–29	223	96.1	9	3.9	Ref				Ref			
30–59	209	95.0	11	5.0	0.564	1.30	0.53	3.21	0.091	3.40	0.84	17.49
> ≥60	18	81.8	4	18.2	**0.009**	**5.51**	**1.54**	**19.65**	**0.002**	**13.93**	**2.66**	**84.21**
Gender	450		24									
Male	144	98.0	3	2.0	Ref				Ref			
Female	306	93.6	21	6.4	0.057	3.29	0.97	11.22	0.057	2.74	0.97	10.44
Born in the same country of residence	450		24									
No	47	97.9	1	2.1	Ref				Ref			
Yes	403	94.6	23	5.4	0.340	2.68	0.35	20.32	0.651	1.46	0.34	13.62
Living status	450		24									
Live with family members	221	96.1	9	3.9	Ref				Ref			
Live with non-family members	136	93.8	9	6.2	0.316	1.63	0.63	4.20	**0.041**	**4.12**	**1.06**	**17.39**
Live alone	93	93.9	6.1	3.9	0.395	1.58	0.55	4.58	0.164	2.19	0.71	6.35
Highest educational/vocational qualification	450		24									
Secondary/Higher Secondary/Grade 7 to 12	178	94.7	10	5.3	Ref				Ref			
Certificate/Diploma/Trade qualifications	77	92.8	6	7.2	0.540	1.39	0.49	3.95	**0.034**	**7.75**	**1.19**	**40.49**
Bachelor/Masters/PhD	195	96.1	8	3.9	0.517	0.73	0.28	1.89	0.756	1.21	0.37	4.08
Current employment condition	450		24									
Unemployed/Housewife/Home maker/Home duties (No source of income)	161	9.8	7	5.1	Ref				Ref			
Jobs affected by COVID-19 (lost job/working hours reduced/afraid of job loss)	8	80.0	2	20.0	**0.047**	**5.75**	**1.03**	**32.25**	0.978	0.98	0.29	3.23
Have an income source (employed/Government benefits)	281	94.9	15	5.1	0.661	1.23	0.49	3.07	0.451	0.65	0.21	2.01
Perceived distress due to change of employment status	375		22									
A little to none	189	94.0	12	6.0	Ref				Ref			
Moderate to a great deal	186	94.9	10	5.1	0.706	0.85	0.36	2.01	0.616	0.79	0.31	1.96
Improved working situation due to change of employment status	384		22									
A little to none	332	94.3	20	5.7	Ref				Ref			
Moderate to a great deal	52	96.3	2	3.7	0.553	0.64	0.15	2.81	0.932	1.06	0.21	3.64
Self-identification as a frontline or essential service worker	450		24									
No	237	94.8	13	5.2	Ref				Ref			
Yes	213	95.1	11	4.9	0.886	0.94	0.41	2.15	0.982	1.01	0.37	2.93
Self-identification as a healthcare worker	450		24									
No	201	93.5	14	6.5	Ref				Ref			
Yes, doctor	63	98.4	1	1.6	0.157	0.23	0.03	1.77	0.345	0.42	0.04	2.41
Yes, nurse	15	83.3	3	16.7	0.126	2.87	0.74	11.11	0.453	1.87	0.34	9.02
Yes, other healthcare worker	171	96.6	6	3.4	0.169	0.50	0.19	1.34	0.121	0.41	0.13	1.27
COVID-19 impacted financial situation	450		24									
No impact	349	95.9	15	4.1	Ref				Ref			
Yes, impacted positively	40	93.0	3	7.0	0.395	1.75	0.48	6.29	0.184	2.48	0.61	7.85
Yes, impacted negatively	61	91.0	6	9.0	0.099	2.29	0.86	6.13	0.163	2.30	0.70	6.66
Affected by the change in financial situation	450		24									
Not at all	188	94.9	10	5.1	Ref				Ref			
Unsure	51	96.2	2	3.8	0.700	0.74	0.16	3.47	0.940	0.94	0.16	3.76
Somewhat	124	95.4	6	4.6	0.858	0.91	0.32	2.57	0.896	0.93	0.31	2.63
A great extent	36	92.3	3	7.7	0.511	1.57	0.41	5.98	0.340	1.94	0.46	6.64
Co-morbidities	450		24									
No	320	95.0	17	5.0	Ref				Ref			
Psychiatric/Mental health problem	33	94.3	2	5.7	0.864	1.14	0.25	5.16	0.429	1.84	0.34	6.72
Other co-morbidities^*^	97	95.1	5	4.9	0.954	0.97	0.35	2.70	0.590	1.33	0.44	3.55
Co-morbidities	450		24									
No	326	97.9	7	2.1	Ref				Ref			
Single co-morbidity	89	90.8	9	9.2	**0.003**	**4.71**	**1.71**	**13.00**	**0.001**	**5.76**	**2.01**	**17.44**
Multiple co-morbidities	35	81.4	8	18.6	**<0.001**	**10.65**	**3.64**	**31.12**	**<0.001**	**9.48**	**2.89**	**32.19**
Perceived status of own mental health	450		24									
Good to Excellent	350	97.2	10	2.8	Ref				Ref			
Poor to Fair	100	87.7	14	12.3	**<0.001**	**4.90**	**2.11**	**11.37**	**<0.001**	**5.83**	**2.41**	**15.02**
Smoking	450		24									
Never smoker	392	94.5	23	5.5	Ref				Ref			
Ever smoker (Daily/Nondaily/ Ex)	58	98.3	1	1.7	0.235	0.29	0.04	2.22	0.167	0.35	0.04	1.46
Increased smoking over the last 6 months	58		1									
No	35	100.0	0	0.0	Ref				Ref			
Yes	23	95.8	1	4.2	0.361	4.53	0.18	116.04	0.334	3.48	0.27	276.80
Current alcohol drinking (last 4 weeks)	450		24									
No	258	94.2	16	5.8	Ref				Ref			
Yes	192	96.0	8	4.0	0.370	0.67	0.28	1.60	0.377	0.68	0.27	1.59
Increased alcohol drinking over the last 6 months	192		8									
No	147	95.5	7	4.5	Ref				Ref			
Yes	45	97.8	1	2.2	0.481	0.47	0.06	3.90	0.928	0.92	0.10	4.72
Contact with known/suspected case of COVID-19	450		24									
No	230	95.8	10	4.2	Ref				Ref			
Unsure	50	98.0	1	2.0	0.464	0.46	0.06	3.68	0.942	0.94	0.10	4.36
Yes, had indirect contact	70	89.7	8	10.3	**0.050**	**2.63**	**1.00**	**6.92**	0.057	2.64	0.97	7.07
Yes, provided direct care	100	95.2	5	4.8	0.803	1.15	0.38	3.45	0.513	1.47	0.44	4.46
Experience related to COVID-19 pandemic	450		24									
No known exposure to COVID-19	322	96.4	12	3.6	Ref				Ref			
Treated in hospital / Ordered to quarantine/ Tested positive / Lived with someone who had Covid	112	91.1	11	8.9	**0.025**	**2.64**	**1.13**	**6.14**	**0.021**	**3.09**	**1.19**	**8.12**
Traveled overseas and had to quarantine	16	94.1	1	5.9	0.630	1.68	0.21	13.71	0.149	4.95	0.49	26.65
Self-identification as a patient (visited a healthcare provider in the last 6 months)	450		24									
No	244	97.6	6	2.4	Ref				Ref			
Yes	206	92.0	18	8.0	**0.008**	**3.55**	**1.39**	**9.12**	**0.024**	**2.81**	**1.14**	**7.77**
Level of psychological distress (K10 categories)	450		24									
Low (score 10–15)	91	97.8	2	2.2	Ref							
Moderate to Very High (score 16–50)	359	94.2	22	5.8	0.170	2.79	0.64	12.08	0.162	2.61	0.71	14.59
Level of coping (BRCS categories)	450		24									
Low resilient coping (score 4–13)	147	93.0	11	7.0	Ref							
Medium to high resilient coping (score 14–20)	303	95.9	13	4.1	0.187	0.57	0.25	1.31	0.190	0.56	0.23	1.35
Healthcare services used to overcome COVID-19 related stress in the last 6 months	450		24									
No	433	96.4	16	3.6	Ref							
Yes	17	68.0	8	32.0	**<0.001**	**12.74**	**4.79**	**33.84**	**<0.001**	**15.26**	**4.88**	**48.84**

*Data are presented as number (n) and percentage (%). P-values ≤ of 0.05 were considered statistically significant*.

*ORs, Odds Ratio; AOR, Adjusted Odds Ratio; CI, Confidence Interval; Ref, Reference category; COVID-19, Coronavirus Disease 19; K-10, Kessler Depression Scale; FCV, Fear of Coronavirus-19 Scale; BRCS, Brief Resilient Coping Scale*.

* *Adjusted for Age, Gender, born in Germany, living status, employment status and level of education*.

*Bold indicated statistical significance*.

### Coping Strategies

Multivariate analyses revealed that having an income source and being a healthcare worker were associated with higher levels of coping. Conversely, higher levels of education, distress due to change in employment, worsened financial situation due to the pandemic, and perceived status of mental health as poor to fair were the factors that predicted lower levels of coping amongst the study participants (Table 4).

**Table 4:** Factors associated with coping among the study population (based on BRCS scoring).

**Characteristics**	**Low levels of coping**		**High levels of coping**		**Unadjusted analysis**			**Adjusted analysis**				
	**n**	**%**	**n**	**%**	**p**	**ORs**	**95% CIs**		**p**	**AORs**	**95% CIs**
**Age groups**	**158**		**316**									
18–29	81	34.9	151	65.1	Ref				Ref			
30–59	71	32.3	149	67.7	0.553	1.13	0.76	1.66	0.830	0.94	0.52	1.69
>60	6	27.3	16	72.7	0.472	1.43	0.54	3.80	0.687	1.25	0.43	3.66
**Gender**	**158**		**316**									
Male	45	30.6	102	69.4	Ref				Ref			
Female	113	34.6	214	65.4	0.400	0.84	0.55	1.27	0.547	0.87	0.56	1.36
**Born in the same country of residence**	**158**		**316**									
No	18	37.5	30	62.5	Ref				Ref			
Yes	140	32.9	286	67.1	0.519	1.23	0.66	2.28	0.788	1.09	0.57	2.11
**Living status**	**158**		**316**									
Live with family members	69	30.0	161	70.0	Ref				Ref			
Live with non-family members	48	33.1	97	66.9	0.528	0.87	0.55	1.35	0.753	1.11	0.59	2.06
Live alone	41	41.4	58	58.6	**0.045**	**0.61**	**0.37**	**0.99**	0.063	0.61	0.36	1.03
**Highest educational/vocational qualification**	**158**		**316**									
Secondary/Higher Secondary/Grade 7 to 12	61	32.4	127	67.6	Ref				Ref			
Certificate/Diploma/Trade qualifications	33	39.8	50	60.2	0.245	0.73	0.43	1.24	**0.013**	**0.43**	**0.22**	**0.83**
Bachelor/Masters/PhD	64	31.5	139	68.5	0.846	1.04	0.68	1.60	**0.034**	**0.53**	**0.29**	**0.95**
**Current employment condition**	**158**		**316**									
Unemployed/Housewife/Home maker/Home duties (No source of income)	73	43.5	95	56.5	Ref				Ref			
Jobs affected by COVID-19 (lost job/working hours reduced/afraid of job loss)	4	40.0	6	60.0	0.831	1.15	0.31	4.24	0.525	1.56	0.40	6.08
Have an income source (employed/Government benefits)	81	27.4	215	72.6	**<0.001**	**2.04**	**1.37**	**3.04**	**<0.001**	**3.33**	**1.90**	**5.87**
**Perceived distress due to change of employment status**	**116**		**236**									
A little to none	53	26.4	148	73.6	Ref				Ref			
Moderate to a great deal	81	41.3	115	58.7	**0.002**	**0.51**	**0.33**	**0.78**	**0.003**	**0.51**	**0.33**	**0.80**
**Improved working situation due to change of employment status**	**140**		**266**									
A little to none	116	33.0	236	67.0	Ref				Ref			
Moderate to a great deal	24	44.4	30	55.6	0.1	0.61	0.34	1.10	0.189	0.67	0.36	1.22
**Self-identification as a frontline or essential service worker**	**158**		**316**									
No	97	38.8	153	61.2	Ref				Ref			
Yes	61	27.2	163	72.8	**0.008**	**1.69**	**1.15**	**2.50**	0.370	1.26	0.76	2.08
**Self-identification as a healthcare worker**	**158**		**316**									
No	90	41.9	125	58.1	Ref				Ref			
Yes, doctor	24	37.5	40	62.5	0.534	1.20	0.68	2.13	0.482	0.76	0.36	1.63
Yes, nurse	5	27.8	13	72.2	0.249	1.87	0.64	5.44	0.534	1.45	0.45	4.64
Yes, other healthcare worker	39	22.0	138	78.0	**<0.001**	**2.55**	**1.63**	**3.98**	**0.016**	**1.91**	**1.13**	**3.24**
**COVID-19 impacted financial situation**	**158**		**316**									
No impact	114	31.3	250	68.7	Ref				Ref			
Yes, impacted positively	14	32.6	29	67.4	0.869	0.95	0.48	1.86	0.669	0.86	0.43	1.73
Yes, impacted negatively	30	44.8	37	55.2	**0.033**	**0.56**	**0.33**	**0.96**	**0.023**	**0.51**	**0.29**	**0.91**
**Affected by the change in financial situation**	**145**		**275**									
Not at all	61	30.8	137	69.2	Ref				Ref			
Unsure	23	43.4	30	56.6	0.087	0.58	0.31	1.08	0.091	0.57	0.29	1.10
Somewhat	37	28.5	93	71.5	0.650	1.12	0.69	1.82	0.843	1.05	0.63	1.75
A great extent	24	61.5	15	38.5	**<0.001**	**0.28**	**0.14**	**0.57**	**<0.001**	**0.23**	**0.11**	**0.47**
**Co-morbidities**	**158**		**316**									
No	111	32.9	226	67.1	Ref				Ref			
Psychiatric/Mental health problem	17	48.6	18	51.4	0.067	0.52	0.26	1.05	0.061	0.49	0.24	1.03
Other co-morbidities^*^	30	29.4	72	70.6	0.504	1.18	0.73	1.91	0.954	0.99	0.60	1.63
**Co-morbidities**	**158**		**316**									
No	102	30.6	231	69.4	Ref				Ref			
Single co-morbidity	38	38.8	60	61.2	0.131	0.70	0.44	1.11	0.110	0.66	0.40	1.10
Multiple co-morbidities	18	41.9	25	58.1	0.140	0.61	0.32	1.17	0.144	0.59	0.29	1.20
**Perceived status of own mental health**	**158**		**316**									
Good to Excellent	87	24.2	273	75.8	Ref				Ref			
Poor to Fair	71	62.3	43	37.7	**<0.001**	**0.19**	**0.12**	**0.30**	**<0.001**	**0.20**	**0.13**	**0.33**
**Smoking**	**158**		**316**									
Never smoker	133	32.0	282	68.0	Ref				Ref			
Ever smoker (Daily/Nondaily/ Ex)	25	42.4	34	57.6	0.117	0.64	0.37	1.12	0.110	0.62	0.34	1.12
**Increased smoking over the last 6 months**	**25**		**34**									
No	16	45.7	19	54.3	Ref				Ref			
Yes	9	37.5	15	62.5	0.531	1.40	0.49	4.05	0.349	1.99	0.47	8.38
**Current alcohol drinking (last 4 weeks)**	**158**		**316**									
No	89	32.5	185	67.5	Ref				Ref			
Yes	69	34.5	131	65.5	0.645	0.91	0.62	1.34	0.375	0.83	0.55	1.25
**Increased alcohol drinking over the last 6 months**	**69**		**131**									
No	52	33.8	102	66.2	Ref				Ref			
Yes	17	37.0	29	63.0	0.690	0.87	0.44	1.73	0.423	0.74	0.35	1.55
**Contact with known/suspected case of COVID-19**	**158**		**316**									
No	85	35.4	155	64.6	Ref				Ref			
Unsure	24	47.1	27	52.9	0.121	0.62	0.34	1.14	0.153	0.64	0.34	1.18
Yes, had indirect contact	19	24.4	59	75.6	0.072	1.70	0.95	3.04	0.077	1.70	0.95	3.06
Yes, provided direct care	30	28.6	75	71.4	0.215	1.37	0.83	2.26	0.290	1.32	0.79	2.20
**Experience related to COVID-19 pandemic**	**158**		**316**									
No known exposure to COVID- 19	116	34.7	218	65.3	Ref				Ref			
Treated in hospital / Ordered to quarantine/ Tested positive / Lived with someone who had Covid-19	38	30.9	85	69.1	0.442	1.19	0.76	1.86	0.378	1.23	0.78	1.93
Traveled overseas and had to quarantine	4	23.5	13	76.5	0.348	1.73	0.55	5.42	0.278	1.92	0.59	6.20
**Self-identification as a patient (visited a healthcare provider in the last 6 months)**	**158**		**316**									
No	79	31.6	171	68.4	Ref				Ref			
Yes	79	35.3	145	64.7	0.398	0.85	0.58	1.24	0.446	0.86	0.58	1.27
**Level of fear of COVID-19 (FCV- 19S categories)**	**158**		**316**									
Low (score 7–21)	147	32.7	303	67.3	Ref				Ref			
High (score 22–35)	11	45.8	13	54.2	0.187	0.57	0.25	1.31	0.201	0.55	0.22	1.38
**Level of distress K-10 Score categories)**	**158**		**316**									
Low (score 10–15)	24	25.8	69	74.2	Ref				Ref			
Moderate to Very High (score 16–50)	134	35.2	247	64.8	0.087	0.64	0.39	1.07	0.130	0.64	0.36	1.14
**Healthcare services used to overcome COVID-19 related stress in the last 6 months**	**158**		**316**									
No	146	32.5	303	67.5	Ref				Ref			
Yes	12	48.0	13	52.0	0.115	0.52	0.23	1.17	0.184	0.55	0.23	1.33

*Data are presented as number (n) and percentage (%). P ≤ of 0.05 were considered statistically significant*.

*ORs, Odds Ratio; AOR, Adjusted Odds Ratio; CI, Confidence Interval; Ref, Reference category; COVID-19, Coronavirus Disease 19; K-10, Kessler Depression Scale; FCV, Fear of Coronavirus-19 Scale; BRCS, Brief Resilient Coping Scale*.

* *Adjusted for Age, Gender, Born in Germany, living status, employment status and level of education*.

*Bold indicated statistical significance*.

### Association Between Psychological Distress, Coping and Fear of COVID-19

The K-10 distress score correlated significantly with the FCV-19S score (spearman's r = 0.331, *p* < 0.001), the BRCS score showed an inverse relationship with the distress and fear scores (spearman's r = −0.276 and – 0.173, *p* < 0.001). People with higher distress had higher levels of fear of COVID-19 and lower coping. On the other hand, people with better coping had lower distress and fear of COVID-19 (Table 5).

**Table 5:** Association between psychological distress, coping and fear of COVID-19 using spearman's partial rank correlation.

**Variables**	**Distress**	**Fear of COVID**	**Coping**
Distress	1	0.331^*^	−0.276^*^
Fear of COVID		1	−0.173^*^
Coping			1

*Controlling for Age, Gender, born in Germany, living status, employment status and level of education*.

* *Significant at p < 0.001 level*.

## Discussion

Moderate to very high levels of psychological distress were associated with being a female, living alone, suffering employment change or worsening the financial situation, and poor mental health, smoking and alcohol consumption. Higher levels of fear of COVID-19 were markable in people of ≥60 years, or those with comorbidities or poor mental health. Having an income source and being a healthcare worker was associated with higher levels of coping.

During the COVID-19 pandemic, the course of psychological disturbances which were associated with psychological distress, fear, and coping strategies among the community members including healthcare workers across the world were well-studied (25). Our study can be seen as a supplement to a global cross-sectional study involving 17 countries (10). The same online instruments were used like the prior global study led by the last author (MAR), but the current study adapted German language. In this study, more females participated than males, which was in line with other similar German studies (16, 26–28) in the first wave of the pandemic, and also supports an Australian (10), Egyptian (15), Bangladeshi (14), Malaysian (13), and global study (10). A possible explanation might be that women were more inclined to share their experiences by participating in the study or women were more impacted due to the pandemic, lockdown or financially that prompted them to participate in the study. The mean age (33.6 years) showed that the study participants of this study were younger than those who were included in similar German studies (16, 26, 27).

For the issue of psychological distress in this study, there were more participants with moderate to very high distress, which was in line with the results of previous studies conducted in Germany (27), Australia (10), Malaysia (13), Bangladesh (14), Hong Kong (19) and globally (10), as well as other studies (28–31). Furthermore, similar to this study, previous studies (10, 13, 14, 27) also reported that females and younger respondents had higher psychological distress compared to the reference group. A previous study showed that women seemed to be more impacted by the pandemic in terms of wellbeing than men (32). According to the findings of this study, the common factors associated with moderate to very high levels of psychological distress were being females, those with change in the employment status, and worsening the financial situation, which was supported by earlier evidence (10, 13, 14). Similarly, Hetkamp and Schweda (33) found that respondents reported reduced sleep quality and moderate generalized anxiety and psychological burdens. A possible explanation could be that participants might experience crucial interference with their everyday lives, which was likely to increase psychological distress while the accessibility of conventional mental health care was limited (25). It could also be assumed that uncertainties about the novel coronavirus, its progression, and variable nature of pandemic, and availability and access to the varied range of evidence also could contribute to the report of various country-wise reports of moderate to a high level of psychological stress. There was also a higher correlation between potential contact with COVID-19 cases, whether direct or indirect, experience with the pandemic, and healthcare use to overcome pandemic stress.

Regarding the issue of fear in this study, there were more participants with low fear, which supports studies conducted in Bangladesh (14), Australia (10), Malaysia (13), and globally (10). That indicated habituation to the threatening situation of the pandemic. However, generalized anxiety could remain elevated over time due to the ongoing nature of pandemic (33). Similarly, a largescale German study among 3,500 randomly selected participants reported mental health (anxiety, depression) impact shortly after the lockdown came into effect (34). This study identified the factors associated with higher fear of COVID-19, which were similar as reported in the earlier studies: being female, and middle-aged, or over 60 (10, 13, 14). Being born in the same country of residence, and having at least a trade/certificate/diploma or bachelor degree were associated with higher levels of fear in this study, which were similar to the study conducted in Bangladesh (14).

Regarding the issue of coping in this study, there were more participants with high levels of coping, which is supported by the previous Malaysian (13) and the global study (10). High resilience coping could be explained by the long period of pandemic in Germany. Having an income source and being a healthcare worker were associated with higher levels of coping, findings of which were different compared to the previous studies (10, 13, 14). Finally, results showed that the COVID-19 pandemic and subsequent lockdown measures in early 2020 might slow the spread of the virus. However, those restrictions forced a sudden and dramatic change to the daily routines of community people, although not all individuals were impacted in the similar way. Some situational factors such as occupation, family status, financial and health impact, personality traits could influence individuals' experience during the ongoing COVID crisis in Germany (35).

This study had few limitations. The participants were included from the Ulm region in Southern Germany, which limits the generalizability across the whole German territory. Furthermore, it wasn't possible to exclude more responses from distressed individuals than non-distressed individuals, potentially resulting in selection bias. Finally, the study findings were limited to individuals who could access to online platforms in order to participate; therefore, there was limited generalizability due to the focus to internet-literate people. However, due to the lockdown measures applied during data collection, an online survey was the only available option to perform this study. One of the most crucial points in our study was collecting the targeted sample size during the pandemic lockdown period. Lastly, this study was the only German study that assessed the factors associated with psychological distress, fear, and coping strategies during the second and third waves of the COVID-19 pandemic. The data collection period coincided with the transition between the second and third waves in Germany, therefore, it was also not unlikely to have increased prevalence of psychological distress amongst the participants who participated in this study.

## Conclusions

This study identified levels of psychological distress, fear and coping amongst the community members during the COVID-19 pandemic in the Ulm region in Southern Germany. In addition, several factors and risk groups that were associated with those outcomes, were identified. The identified higher risk groups should be prioritized for receiving mental health support from the relevant healthcare providers such as family physicians and psychiatrists, and automated follow-up reminders could be sent through text messages which would prevent further deterioration of mental health conditions.

## Data Availability Statement

The original contributions generated for this study are included in the article/Supplementary Material, further inquiries can be directed to the corresponding author/s.

## Ethics Statement

The studies involving human participants were reviewed and approved by the Research Ethical Committee (REC) of the Ulm University (Ethical Approval Number 448/20 – FSt/Sta). The patients/participants provided their written informed consent to participate in this study.

## Author Contributions

ME had substantial contribution to the conception or design of the study, data collection, and scientific writing of the manuscript. CS-L contributed to the conception and revised the manuscript critically. XW coordinated data collection. KD performed the statistical analysis. MK took part in scientific writing. ER, RA, MD, MG, and BC revised the manuscript critically for important intellectual content. SA, BB, and WC provided critical feedback on the narrative structure and methods and results. MAR conceptualized the study, coordinated data collection, provided critical feedback, and revised the manuscript. All authors contributed to the article and approved the submitted version.

## Conflict of Interest

The authors declare that the research was conducted in the absence of any commercial or financial relationships that could be construed as a potential conflict of interest.

## Publisher's Note

All claims expressed in this article are solely those of the authors and do not necessarily represent those of their affiliated organizations, or those of the publisher, the editors and the reviewers. Any product that may be evaluated in this article, or claim that may be made by its manufacturer, is not guaranteed or endorsed by the publisher.
