# Supplementary material for: Psychological Distress, Fear and Coping Strategies During the Second and Third Waves of the COVID-19 Pandemic in Southern Germany
Source: Front Psychiatry. 2022 Apr 25;13:860683. doi: 10.3389/fpsyt.2022.860683 (PMC9082598; doi:10.3389/fpsyt.2022.860683)
Supplement: Supplementary file 1 [file Data_Sheet_1.PDF]

COVID-19 - Psychischer Stress und Bewältigungsstrategien bei Patienten, Gesundheitspersonal im Kontakt mit COVID-19 und weiteren Mitglieder der Gesellschaft: Eine online Befragung.

Sehr geehrte Studienteilnehmerin, sehr geehrter Studienteilnehmer,

Wir möchten Sie bitten, an der folgenden Befragung teilzunehmen:

In diesem Projekt werden die psychologischen Auswirkungen von Beschäftigten im Gesundheitswesen, ihren Patienten und Menschen in der Gesellschaft in einer Reihe von Ländern weltweit in Bezug auf Angstzustände, Depressionen, Stress und Bewältigungsstrategien untersucht. Die Befragung in deutscher Sprache richtet sich nur an Teilnehmer/innen, die derzeit in Deutschland leben und genügend Deutschkenntnisse besitzen um die gestellten Fragen zu verstehen. Mitarbeiter und Patienten im Gesundheitswesen, sowie sonstige Mitglieder der Gesellschaft können sich entscheiden, über verschiedene soziale Medien und Online-Netzwerke an dieser Studienumfrage teilzunehmen. Die Studienteilnehmer werden gebeten, einen strukturierten Online-Fragebogen auszufüllen, welcher zur Bewertung der psychologischen Auswirkungen und deren Bewältigungsstrategien im Zusammenhang mit COVID-19 dient. Die Antworten werden anonym gesammelt.

Die Coronavirus-Krankheit-2019 (COVID-19) hat durch die rasche Ausbreitung unser Leben auf der ganzen Welt verändert. Durch die steigenden Infektionszahlen und Todesfälle weltweit sowie durch wirtschaftliche Auswirkungen auf unsere Gesellschaft nimmt die Angst vor dem Coronavirus stetig zu. Das Gefühl der Ungewissheit, wie lange die Situation andauern wird, die soziale Distanzierung, die Sorge vor Arbeitslosigkeit sowie die Angst, dass man selbst oder Familienmitglieder an COVID-19 erkranken, ist weit verbreitet. Es bestehen zusätzliche Befürchtungen, dass die Gesundheitssysteme überlastet werden könnten und den Betroffenen keine angemessene Gesundheitsversorgung zur Verfügung stehen könnte. Es gibt zunehmende Bedenken, dass sich der verursachte Stress und die erwähnte Angst auf die Psyche negativ auswirken können. Infolge der zunehmenden Anzahl bestätigter Infektionen und Todesfälle durch COVID-19 wurden sowohl Mitarbeiter des Gesundheitswesens als auch Patienten und weitere Mitglieder der Gesellschaft mit übermäßigen Stress, Angstzuständen und Depressionen im Alltag konfrontiert. Die COVID-19-Pandemie könnte zudem die Beschäftigten im Gesundheitswesen in eine beispiellose Situation bringen, in der sie großem Stress ausgesetzt werden dadurch, dass sie gelegentlich gezwungen sein könnten schwierige Entscheidungen (z.B. Infolge einer Triage-Entscheidung) zu treffen. Eine neue Herausforderung könnte die persönliche oder telemedizinische Behandlung von Patienten mit eingeschränkten Ressourcen darstellen.

ERREICHBARKEIT DES STUDIENARZTES:

Sollten während des Verlaufes des Forschungsprojektes (Ausfüllens eines Fragebogens) Fragen auftauchen, so können Sie täglich an Werktagen (zwischen 8:00h und 17:00h) folgende Ansprechpartner erreichen:

Studienleiter Prof. Dr. med. C. Schönfeldt-Lecuona unter der Telefonnummer 0731 500-61411, oder den stellvertretenden Studienleiter Mohamed Elsayed unter 0731 500-61579.

VERSICHERUNG:

Während der Teilnahme an dem Forschungsprojekt genießen Sie Versicherungsschutz. Das Universitätsklinikum Ulm und dessen an der Studie mitwirkende Mitarbeiter (Studienärzte, sonstiges Personal) sind haftpflichtversichert für den Fall, dass Sie durch deren Verschulden einen Schaden erleiden. Einen Schaden, der Ihrer Meinung nach auf die Untersuchung zurückzuführen ist, melden Sie bitte unverzüglich dem Studienarzt/-leiter.

SCHWEIGEPFLICHT/DATENSCHUTZ:

Alle Personen, welche Sie im Rahmen dieses Projektes betreuen, unterliegen der Schweigepflicht und sind auf das Datengeheimnis verpflichtet. Die studienbezogenen Untersuchungsergebnisse sollen in anonymisierter Form in wissenschaftlichen Veröffentlichungen verwendet werden.Die Daten werden im Google-Formular gespeichert und nur der Projektleiter und der Ko-Leiter, sowie die Ko-Autoren der Studienteams haben Zugriff auf die Daten. Die Datenerfassung ist vollständig anonym und im Google-Formular sind keine IP-Adressen hinterlegt. Die Daten sind auf dem Google-Formular mit einem Kennwort gesichert. Soweit es zur Kontrolle der korrekten Datenerhebung erforderlich ist, dürfen autorisierte Personen (z.B. durch den Auftraggeber, durch die Universität) Einsicht in die studienrelevanten Teile der Krankenakte nehmen.

Sofern zur Einsichtnahme autorisierte Personen nicht der obengenannten ärztlichen Schweigepflicht unterliegen, stellen personenbezogene Daten, von denen sie bei der Kontrolle Kenntnis erlangen, Betriebsgeheimnisse dar, die geheim zu halten sind. Die in diesem Projekt für die Datenverarbeitung verantwortliche Person ist:

Mohamed Elsayed, Facharzt für Psychiatrie und Psychotherapie,
Klinik für Psychiatrie und Psychotherapie III Leimgrubenweg 12-14
89075 Ulm
Telefon: 0731 500-61579
E-Mail: mohamed.elsayed@uni-ulm.de.

Bei Fragen zur Nutzung oder Verarbeitung Ihrer Daten wenden Sie sich bitte an den/die:
Datenschutzbeauftragte/n des lokalen Studienzentrums
(a) Universität Ulm: Universität Ulm, Helmholtzstr. 16, 89081 Ulm, Tel.Nr.: 07542 / 949 21 09, E-Mail: dsb@uni-ulm.de
(b) Uniklinikum Ulm: Klinikumsverwaltung, Albert-Einstein-Allee 29, 89081 Ulm, Tel.Nr.: 0731 / 500-69290, E-Mail: dsb.ukl@uniklinik-ulm.de

Falls Sie Bedenken oder Beschwerden hinsichtlich der Verarbeitung Ihrer Daten haben, wenden Sie sich bitte an die Datenschutz-Aufsichtsbehörde Ihres Studienzentrums. Die entsprechenden Kontaktdaten finden Sie auf der Internetseite des Landesbeauftragten für Datenschutz und Informationsfreiheit Baden-Württemberg:
https://www.baden-wuerttemberg.datenschutz.de/dsb-online-melden/

Online-Erklärung des Teilnehmers:

- Ich habe die Erklärung zu den Informationen in einfacher Sprache gelesen und verstanden.
- Ich verstehe die Zwecke, Verfahren und Risiken der im Projekt beschriebenen Forschung.
- Ich hatte Gelegenheit Fragen zu stellen und bin mit den Antworten, die ich erhalten habe, zufrieden.
- Ich bin damit einverstanden, wie beschrieben an diesem Forschungsprojekt teilzunehmen, und verstehe, dass es mir jederzeit gestattet ist, während des Ausfüllens des Online- Fragebogens zurückzutreten.

Ich bin einverstanden, dass die Daten anonym gesammelt werden und keine identifizierenden Informationen für diese Online-Umfrage beigefügt werden. Wenn die Einwilligung nach der Sammlung und Verarbeitung der Daten widerrufen wird, können die Daten nicht aus der Auswertung zurückgezogen werden.

- Ich bin damit einverstanden, dass von mir oder mit meiner Erlaubnis während des Projekts bereitgestellte Informationen in eine Abschlussarbeit, Präsentation und Veröffentlichung in Fachzeitschriften aufgenommen werden können.

Wenn Sie sich während des Ausfüllens des Studienfragebogens sehr belastet oder verzweifelt fühlen, werden die Informationen zur Kontaktaufnahme mit den länderspezifischen Hotlines in das PLIS aufgenommen.

Das Landratsamt Alb-Donau-Kreis hat eine Telefon-Hotline für Rat suchende Bürgerinnen und Bürger aus dem Alb-Donau-Kreis und dem Stadtkreis Ulm eingerichtet (Telefonnummer 0731/185-1050).
Alternativ können Sie den Studienarzt und Projekt Ko-Leiter kontaktieren, Herrn Mohamed Elsayed, Facharzt für Psychiatrie und Psychotherapie, Tel: 0731-500 61500 E-Mail: Mohamed.Elsayed@uni-ulm-de, oder die Psychiatrische Institutsambulanz, Klinik für Psychiatrie III, Universitätsklinikum Ulm, (Tel: 0731-500 61500).

- Wenn ein Teilnehmer die Ergebnisse der Studie wissen möchte, kann der Studienarzt über das Informationsblatt angegebene E-Mail kontaktiert werden, und eine einseitige Zusammenfassung wird geteilt (E-Mail: Mohamed.Elsayed@uni-ulm.de)

Klicken Sie auf Optionen

- Ich bin damit einverstanden, an dieser Studie teilzunehmen: Es wird mit dem nächsten Bildschirm des Fragebogens fortgefahren

ODER

- Ich bin nicht einverstanden, an dieser Studie teilzunehmen: dem Befragten wird gedankt und der Bildschirm wird geschlossen.

Vielen Dank für die Bereitschafft an diesem Projekt teilzunehmen

1. Stimmen Sie der Teilnahme an dieser Studie zu? *

Ich bin damit einverstanden, an dieser Studie teilzunehmen
Ich bin nicht einverstanden, an dieser Studie teilzunehmen

Einverständniserklärung

2. Sind Sie mindestens 18 Jahre alt? *

Ja
Nein

Studien Fragebogen

3. Wie alt sind Sie? *

4. In welchem Land leben Sie? *

5. Wurden Sie in diesem Land geboren? *
Wenn die Antwort anders ist (Bitte schreiben Sie den Namen des Landes, in dem Sie geboren wurden)

- Ja
- **Andere**

6. Leben Sie *

- Alleine
- Mit Ihrem Partner
- Mit Ihrem Partner und Ihren Kindern
- Mit Kindern und ohne Partner
- In einer Wohngemeinschaft mit Nicht-Familienmitgliedern
- Keine Aussage trifft zu

7. Ihr Geschlecht: *

- Weiblich
- Männlich
- Andere
- Keine Angabe

8. Wie lautet die höchste Bildungs- oder Berufsqualifikation, die Sie abgeschlossen haben? *

- Grundschule
- Hauptschulabschluss
- Mittlere Reife / Realschulabschluss
- Gymnasium
- Berufsausbildung / Lehre (z.B. Friseur, Elektriker, Klempner)
- Aufstiegsweiterbildung (z.B. Techniker, Meister)
- Universitätsabschluss / Hochschulabschluss

9. Was sind Ihre aktuellen Arbeitsbedingungen? (Kreuzen Sie die zutreffenden Felder an) *

- [ ] Ich habe einen Beruf und arbeite aufgrund der Pandemie im Homoffice
- [ ] Ich habe einen Beruf, für den ich trotz der COVID-19-Pandemie das Haus verlassen muss
- [ ] Ich habe einen Beruf und befinde mich wegen der COVID-19-Pandemie in Kurzarbeit
- [ ] Ich habe meinen Beruf wegen der COVID-19-Pandemie verloren
- [ ] Ich war auch schon vor der COVID-19-Pandemie arbeitslos/arbeitssuchend
- [ ] Ich bin Student, aber mein Studium findet wegen der COVID-19-Pandemie online statt
- [ ] Ich bin Student, aber mein Studium wurde wegen der COVID-19-Pandemie pausiert
- [ ] Ich bin berentet
- [ ] Ich gehe weiterhin trotz der COVID-19-Pandemie nicht entgeltlichen Arbeiten nach (z.B. Haushaltsführung, Ehrenamt)
- [ ] Keine Aussage trifft zu

10. Hat es eine Änderung Ihrer Beschäftigungsbedingungen (Hauptbeschäftigung: Arbeitsstelle, Studium) aufgrund der COVID-19-Pandemie gegeben? *

- Ja
- Nein

11. Falls ja: Hat diese Änderung der Beschäftigungsbedingungen aufgrund der COVID-19-Pandemie Ihre Arbeitssituation verschlechtert?

- Sehr stark
- Stark
- Eher Mäßig
- Etwas
- Überhaupt nicht

12. Hat diese Änderung der Beschäftigungsbedingungen aufgrund der COVID-19-Pandemie Ihre Arbeitssituation verbessert?

- Sehr stark
- Stark
- Eher Mäßig
- Etwas
- Überhaupt nicht

13. Was ist / war Ihre berufliche Hauptbeschäftigung? (Bitte geben Sie keine Informationen preis, durch die Sie identifiziert werden könnten) *

14. Werden Sie während der COVID-19-Pandemie als systemrelevante Mitarbeiter oder Dienstleister angesehen? (z.B. Angestellter im Gesundheitswesen, Altenpflege, Supermarktarbeiter, Bankier, Polizist oder Rettungswagen, oder andere wichtige Dienstleistungen) *
(Wenn ja, wählen Sie eine andere, geben Sie bitte Ihre Berufsbezeichnung an.) (Bitte geben Sie keine Informationen an, die Sie identifizieren könnten!)

- Nein
- Ja
- Andere

15. Sind Sie ein Angestellter im Gesundheitswesen? *

- Ja, Arzt/Ärztin
- Ja, Krankenpfleger
- Ja, andere Mitarbeiter des Gesundheitswesens
- Nein

16. Hat sich die COVID-19-Pandemie auf Ihre finanzielle Situation ausgewirkt? *

- Positiv
- Negativ
- Keine Änderung

17. Falls ja, wie hat sich diese Veränderung auf Ihre psychisch Wohlbefinden ausgewirkt?

- Sehr
- etwas
- Unsicher zu diesem Zeitpunkt
- Überhaupt nicht

18. Hat Ihnen Ihr Arzt jemals gesagt, dass Sie eine oder mehrere der folgenden Erkrankungen haben / hatten?
(Zutreffendes ankreuzen)

- [ ] Herzerkrankung
- [ ] Schlaganfall
- [ ] Hoher Blutdruck / Hypertension
- [ ] Hoher Cholesterinspiegel / hohe Triglyceride
- [ ] Diabetes oder hoher Zuckergehalt im Blut oder Urin
- [ ] Krebs
- [ ] Psychisches Gesundheitsproblem
- [ ] Chronische Atemwegserkrankung

19. Rauchen Sie? *

- Ja **(Fahren Sie mit Frage 20 fort)**
- Nein **(Weiter mit Frage 27)**

Fragen zum Rauchen (nur für Raucher)

20. Falls ja, wann nach dem Aufstehen rauchen Sie Ihre erste Zigarette? *

- Nach 5 Minuten
- Nach 6 - 30 Minuten
- Nach 31 - 60 Minuten
- Nach mehr als 60 Minuten

21. Finden Sie es schwierig, an Orten, wo das Rauchen verboten ist, das Rauchen zu unterlassen? *

- Ja
- Nein

22. Auf welche Zigarette würden Sie nicht verzichten wollen? *

- Die erste am Morgen
- Andere

23. Wie viele Zigaretten rauchen Sie im Allgemeinen pro Tag? *

- 31 und mehr
- 21 - 30
- 11 - 20
- Bis 10

24. Rauchen Sie am Morgen im Allgemeinen mehr als am Rest des Tages? *

- Ja
- Nein

25. Kommt es vor, dass Sie rauchen, wenn Sie krank sind und tagsüber im Bett bleiben müssen? *

- Ja
- Nein

26. Haben Sie seit März 2020/Beginn der Pandemie vermehrt geraucht? *

- Ja
- Nein
- Keine Angabe

Studien Fragebogen 2

27. Trinken Sie zurzeit Alkohol? *

- Ja **(Weiter mit Frage 28)**
- Nein **(Weiter mit Frage 41)**
- Keine Angabe **(Weiter mit Frage 41)**

Fragen zum Alkoholkonsum

28. Falls ja, wie oft trinken Sie alkoholische Getränke? *

- Einmal pro Monat oder seltener
- 2- bis 4-mal pro Monat
- 2- bis 3-mal pro Woche
- 4-mal pro Woche oder öfter

29. Wenn Sie Alkohol trinken, wie viele alkoholische Getränke nehmen Sie dann im Schnitt zu sich? Beispiel: Ein alkoholisches Getränk entspricht 0,2 L Wein ODER 0,5 L Bier ODER 2 CL Schnaps/Likör ODER 1 Cocktail *

- 1 oder 2
- 3 oder 4
- 5 oder 6
- 7 bis 9
- 10 oder mehr

30. Wie oft nehmen Sie 5 oder mehr alkoholische Getränke auf einmal zu sich? *

- Nie
- Weniger als einmal pro Monat
- Einmal pro Monat
- Einmal pro Woche
- Täglich oder fast täglich

31. Wie oft konnten Sie im letzten Jahr nicht mit dem Trinken aufhören, nachdem Sie begonnen hatten – obwohl Sie nicht vorhatten so viel zu trinken? *

- Nie
- Weniger als einmal pro Monat
- Einmal pro Monat
- Einmal pro Woche
- Täglich oder fast täglich

32. Wie häufig konnten Sie im letzten Jahr aufgrund von Alkoholkonsum nicht das tun, was eigentlich von Ihnen erwartet wurde, z.B. zur Arbeit gehen, Haushaltstätigkeiten erledigen, eine Verabredung wahrnehmen? *

- Nie
- Weniger als einmal pro Monat
- Einmal pro Monat
- Einmal pro Woche
- Täglich oder fast täglich

33. Wie oft haben Sie im letzten Jahr ein alkoholisches Getränk am Morgen benötigt, um nach einer Nacht mit heftigem Alkoholgenuss in Schwung zu kommen? *

- Nie
- Weniger als einmal pro Monat
- Einmal pro Monat
- Einmal pro Woche
- Täglich oder fast täglich

34. Wie oft hatten Sie im letzten Jahr nach Alkoholkonsum Schuld- oder Reuegefühle? *

- Nie
- Weniger als einmal pro Monat
- Einmal pro Monat
- Einmal pro Woche
- Täglich oder fast täglich

35. Wie oft haben Sie sich im letzten Jahr aufgrund Ihres Alkoholkonsums nicht mehr daran erinnert, was am Abend vorher passiert war? *

- Nie
- Weniger als einmal pro Monat
- Einmal pro Monat
- Einmal pro Woche
- Täglich oder fast täglich

36. Wurden Sie selbst oder jemand anders in Folge Ihres Alkoholkonsums verletzt? *

- Nein
- Ja, aber nicht im letzten Jahr
- Ja, im letzten Jahr

37. Hat sich ein Angehöriger, ein Freund, ein Kollege oder ein Arzt schon einmal besorgt geäußert über Ihren Alkoholkonsum oder Ihnen vorgeschlagen, diesen zu reduzieren? *

- Nein
- Ja, aber nicht im letzten Jahr
- Ja, im letzten Jahr

38. Falls Sie Alkohol trinken, wie oft haben Sie seit März 2020 Alkohol getrunken? *

- Jeden Tag
- 5 Mal pro Woche
- 2-4 Mal pro Woche
- Einmal pro Woche
- Nur am Wochenende
- Zu besonderen Anlässen
- Niemals

39. Haben Sie Ihren Alkoholkonsum seit März 2020 erhöht? *

- Ja
- Nein
- keine Angabe

40. Wie hoch ist der Alkoholgehalt in Ihren Getränken gewöhnlicherweise, wenn Sie Alkohol trinken? *

- 2,5%
- 5,0%
- 10-12,5%
- 40%

Studien Fragebogen 3

41. Hatten Sie zu einem Familienmitglied oder einem Patienten mit festgestelltem oder Verdacht auf COVID-19 direkt oder indirekt Kontakt? *

- Ja, direkter Kontakt
- Ja, indirekter Kontakt
- Unsicher
- Nein

42. Ist Ihnen im Zusammenhang mit der COVID-19-Pandemie eines der folgenden Ereignisse widerfahren? *
(Zutreffendes ankreuzen)

- [ ] Ich wurde wegen COVID-19 in einem Krankenhaus behandelt
- [ ] Ich wurde positiv auf COVID-19 getestet, musste aber nicht ins Krankenhaus
- [ ] Mir wurde aufgrund von COVID-19 eine Quarantäne angeordnet
- [ ] Ich war vor kurzem auf Reisen und befand mich in Quarantäne
- [ ] Jemand, der bei mir lebt, hat oder hatte COVID-19
- [ ] Ich war bisher weder direkt noch indirekt mit COVID-19 in Kontakt und war bisher auch noch nicht in Quarantäne

43. Haben Sie in den letzten 4 Wochen als Patient/in Gesundheitsdienste in Anspruch genommen? *

- Ja
- Nein

44. Wenn ja, welchen Gesundheitsdienst haben Sie in Anspruch genommen?
(Zutreffendes ankreuzen)

- [ ] Besuch bei einem Hausarzt oder einem medizinischen Fachpersonal
- [ ] Telemedizin-Konsultation (online oder per Telefon) mit einem Allgemeinmediziner, Spezialisten oder Angehörigen eines Gesundheitsberufes
- [ ] Krankenhausbesuch für den COVID-19-Test
- [ ] Ich wurde an einer speziellen Test-Stelle auf COVID-19 getestet
- [ ] Anruf bei der nationalen Hotline für symptomatisches Management von COVID-19
- [ ] Notaufnahme eines Krankenhauses
- [ ] Ich war aus anderen Gründen in einem Krankenhaus
- [ ] Keine Aussage trifft zu

45. Generell, wie würden Sie Ihre psychische Gesundheit beurteilen? *

- Ausgezeichnet
- Sehr gut
- Gut
- Mäßig
- Schlecht

46. Die folgenden Fragen beziehen sich auf den Zeitraum seit Beginn der Pandemie im März 2020: *
In den letzten 4 Wochen...

	Niemals	Selten	Manchmal	Sehr oft	Die ganze Zeit
Wie oft haben Sie sich ohne erklärbaren Grund ermüdet gefühlt?					
Wie oft waren Sie nervös?					
Wie oft waren Sie so nervös, dass Sie sich nicht beruhigen konnten?					
Wie oft fühlten Sie sich hoffnungslos?					
Wie oft fühlten Sie sich unruhig oder zappelig?					
Wie oft fühlten Sie sich so unruhig, dass Sie nicht stillsitzen konnten?					
Wie oft fühlten Sie sich sehr deprimiert?					
Wie oft hatten Sie das Gefühl, dass alles anstrengend ist?					
Wie oft waren Sie so traurig, dass Sie nichts aufmuntern konnte?					
Wie oft fühlten Sie sich wertlos?					

47. Wie sehr treffen folgende Aussagen auf Sie zu? *

	Trifft überhaupt nicht auf mich zu	Trifft eher nicht auf mich zu	Neutral	Trifft eher auf mich zu	Trifft sehr gut auf mich zu
Ich habe Angst vor COVID-19.					
Es ist für mich unangenehm, an COVID-19 zu denken.					
Meine Hände werden feucht/kalt, wenn ich an COVID-19 denke.					
Ich habe Angst, mein Leben wegen COVID-19 zu verlieren.					
Beim Ansehen von Nachrichten und Geschichten über COVID-19 in sozialen Medien werde ich nervös oder bekomme Angst.					
Ich kann nicht schlafen, weil ich mir Sorgen wegen COVID-19 mache.					
Mein Herz rast oder klopft, wenn ich daran denke, COVID-19 zu bekommen.					

48. Wie gut kommen Sie mit der COVID-19-Pandemie zurecht? *

	Trifft überhaupt nicht auf mich zu	Trifft eher nicht auf mich zu	Neutral	Trifft eher auf mich zu	Trifft sehr gut auf mich zu
Ich suche nach kreativen Wegen, um schwierige Situationen zu verändern.					
Unabhängig davon, was mit mir passiert, glaube ich, dass ich meine Reaktion darauf kontrollieren kann.					
Ich glaube, ich kann durch den Umgang mit schwierigen Situationen positiv wachsen.					
Ich suche aktiv nach Wegen, um die Verluste zu ersetzen, denen ich im Leben begegne.					

49. Haben Sie etwas anderes getan, um mit Ihrem Stress während der COVID-19-Pandemie umzugehen? (Bitte geben Sie keine Informationen preis, durch die Sie identifiziert werden könnten) *

50. Haben Sie seit März 2020 medizinische Leistungen erhalten, um den mit COVID-19 verbundenen Stress zu überwinden? *

- Ja
- Nein

51. Wenn ja, welchen Gesundheitsdienst haben Sie in Anspruch genommen?

(Zutreffendes ankreuzen)

- [ ] Konsultierte einen Hausarzt
- [ ] Konsultierte einen Psychologen
- [ ] Konsultierte einen Psychiater
- [ ] Verwendete spezielle psychiatrische Einrichtungen (Krankenhaus, kommunale psychiatrische Versorgung, stationäre psychiatrische Versorgung)
- [ ] Verwendete Ressourcen für psychische Gesundheit (Flugblätter, Broschüren, Prospekte und Bücher, die von Mitarbeitern für psychische Gesundheit bereitgestellt und im Krankenhaus verteilt wurden)
- [ ] Verwendete Ressourcen für psychische Gesundheit, die über Medien verfügbar sind (Methoden und Techniken der psychologischen Unterstützung, die von Psychologen über Online-Medien, Fernsehnachrichten oder verschiedene Online- und soziale Netzwerkplattformen bereitgestellt wurden)
- [ ] In Anspruch genommene Unterstützungsdienste für psychische Gesundheit (einschließlich Programm für psychische Gesundheit)

Ende des fragebogens

Wir bedanken uns sehr für Ihre Zeit und Ihre Bemühungen, an dieser wichtigen Studienumfrage teilzunehmen.

Falls Sie sich schlecht oder verzweifelt fühlen sollten, gibt es im Folgenden Informationen über Einrichtungen, an die Sie sich wenden können, um Hilfe zu erhalten:

Das Landratsamt Alb-Donau-Kreis hat eine Telefon-Hotline für Rat suchende Bürgerinnen und Bürger aus dem Alb-Donau-Kreis und dem Stadtkreis Ulm eingerichtet (Telefonnummer 0731/185-1050). Alternativ können Sie den Studienarzt und Projekt Ko-Leiter kontaktieren, Herrn Mohamed Elsayed, Facharzt für Psychiatrie und Psychotherapie, Tel: 0731-500 61500 E-Mail: Mohamed.Elsayed@uni-ulm-de, oder die Psychiatrische Institutsambulanz, Klinik für Psychiatrie III, Universitätsklinikum Ulm (Tel: 0731-500 61500).
